# Indents

**Published:** 1911-07-15

**Authors:** 


### INDENTS.

**☞Brief Articles of Technical or Scientific Value will receive notice and comment. Contributions invited.**

*The Functions of The Nose.* So much, in brief, as to the causes. Let us for a few moments review the functions of the "breath-way," in order that we may have a clear understanding of the evils wrought from obstruction to nasal breathing.

It is elementary to re-state that the functions of the nose may be classed as (1) respiratory, (2) olfactory, (3) auditory, and (4) vocal.

*Respiratory function.* The respiratory function of the nose is the most important. The nasal chambers are more than mere tubes through which air is drawn into the lungs. They produce certain changes in the air which prepare it so that the normal transfusion of oxygen and carbon dioxid may take place through the walls of the air vesicles. The respiratory functions of the nose are fourfold: (*a*) The filtering of germs and dirt from the inspired air; (*b*) the warning of the air; (*c*) the moistening of the air, and (*d*) the destruction of some of the disease-producing bacteria.

*Olfactory function.* That the nose is a filter is made evident by examining the hairs or vibrissae of the vestibule and finding them laden with dirt and bacteria. Those which escape are caught on the mucous membrane lining the nose, and rendered sterile by the inherent sterilizing quality of the nasal secretions.

Experiments show that no matter what the temperature of the inspired air may be, it is raised or lowered to near the body temperature, viz, $98\frac{1}{2}°$ F. Expired air invariably contains more moisture than that which is inspired. This increase in moisture is for the most part added to the air that passes through the nose during inspiration, the erectile tissue of the nose, or "swell bodies," and the serum secreting glands of the nasal mucosa being the principal sources. It is estimated that the total amount of water secreted by the nose and necessary to moisten the inspired air is from 12 to 18 ounces daily, varying with the temperature of the inspired air and the amount of moisture it contains.

*Auditory.* As to the auditory function of the nose, it is important to bear in mind that the Eustachian tube which enters the naso-pharynx serves the purpose of ventilating the middle ear, thus equalizing the atmospheric pressure on the external and internal surfaces of the membrana tympani. Whenever there is partial or complete obstruction to nasal respiration, mouth-breathing is indulged in; the current of air entering the mouth and passing just below the naso-pharynx aspirates some of the air from the naso-pharynx, Eustachian tube, and the cavity of the middle ear, thus diminishing slightly the atmosphere pressure within the middle ear. The effect of this is to cause a sinking-in of the membrana tympani and to blunt somewhat the acuteness of hearing.

*Vocal function.* While sound is produced within the larynx by vibration of the vocal cords, certain of the sounds receive added characteristics from the vibration of the air in its passage through the naso-pharynx and the nasal cavities. Whenever the nose or the naso-pharynx is obstructed, these vibrations are made abnormal or impossible, hence that part of the sound derived from the nasal cavity is absent and the person is said to be "talking through his nose."—*Dr. McConachie—April Cosmos.*

*The Lucas Splint for the Retention of Loose Lower Anterior Teeth.* A modeling compound impression is taken of the labial surfaces of the teeth to be splinted, including within the impression at least one sound tooth on either extremity of the impression. This modeling compound impression will hereafter be called the temporary splint, and is used for holding the teeth in position while they are being prepared for the splint. The temporary splint is removed and trimmed so that its upper surface inclines lingually, and terminates at the incisal edges of the teeth. The temporary splint is then placed in position, and with right angle burs, 1½ mm. in diameter holes are drilled horizontally in the lingual surfaces of the teeth to be splinted. The holes are placed high enough so that they will not touch or irritate the pulp, and low enough to secure a depth of the holes of about 2½ mm. Care should be taken to drill all holes exactly horizontal and parallel with each other in all directions.

A small quantity of low-fusing inlay wax is then placed in each hole, and double headed platinum pins from broken porcelain teeth are heated and inserted into the holes to a depth of 2½ mm. The excess wax which is forced out is then trimmed away, in ordre to expose the clean heads of the pins.

Next the labial plate of a crown and bridge impression tray is trimmed off, leaving only the lingual walls and floor. Using this tray, and with the temporary splint in position, an impression of the lingual surfaces only of the teeth to be splinted. is taken with quick-setting plaster. When the plaster has set, the temporary splint is removed, a blast of hot air is directed against the labial surfaces of the teeth to soften the wax which is holding the pins in position, and the impression is removed horizontally toward the lingual surface. A perfect impression containing all the pins is necessary. The impression is then painted with shellac and sandarac, and a model is run of investment material the same as is used for inlays.

In separating the impression and the model, the impression is shaved down carefully until the heads of the pins are exposed, and then with a fine instrument the plaster is chipped from around the heads of the pins, so that the whole impression may be removed without withdrawing the pins from the model. The model is then trimmed to a size suitable for investing in the casting machine. The wax splint is then built upon the model, care being taken to cover the pins, carving the splint to the desired final size.

The sprue wire is then adjusted in the center of the wax splint. If the splint is to include more than four teeth, or has a decided curvature, wires for vents should be inserted in the extreme ends of the wax splint. The model should be allowed to become watersoaked before it is invested in the casting cup. The model is then invested, and the casting made.

After having been ground, polished, and finished, the splint is ready to be set at the second visit. If the impression and model have been perfect, the splint will fit as accurately as an inlay. Before the splint is permanently set with cement, the inside of the holes in the teeth should be enlarged very slightly by the use of an inverted-cone bur, to allow for better anchorage of the cement.—*J. H. Crawford, in Cosmos.*

